# Prevalence of postpartum depression and associated factors among women in Mbarara and Rwampara districts of south-western Uganda

**DOI:** 10.1186/s12884-021-03967-3

**Published:** 2021-07-12

**Authors:** Catherine Atuhaire, Godfrey Zari Rukundo, Grace Nambozi, Joseph Ngonzi, Daniel Atwine, Samuel Nambile Cumber, Laura Brennaman

**Affiliations:** Faculty of Medicine, Department of Nursing, Mbarara University of Science and Technology (MUST), Mbarara, Uganda

**Keywords:** Postpartum depression, Prevalence, Associated factors, Uganda

## Abstract

**Background:**

Postpartum depression (PPD) is a significant cause of maternal morbidity and has severe consequences on the well-being of mothers, new-borns, families, and communities. PPD reduces the mother’s response to the child’s needs. In severe cases, mothers suffering from PPD are prone to postpartum psychosis, commit suicide and, in rare cases, infanticide. We aimed to determine the prevalence and understand the factors associated with PPD among mothers in southwestern Uganda.

**Methods:**

This was a cross-sectional study between November 2019 and June 2020 among 292 mothers, 6 to 8 weeks’ postpartum. Mothers were selected from three health facilities in southwestern Uganda and enrolled using stratified consecutive sampling. Postpartum depression was clinically diagnosed using the Diagnostic and Statistical Manual of Mental Disorders V. The factors associated with PPD were assessed by using a structured interviewer administered questionnaire. The factors were analyzed using bivariate chi square analyses and multivariate logistic regression.

**Results:**

Overall prevalence of PPD was 27.1% (95% CI: 22.2–32.5). This did not vary by the number of previous births or mode of birth. Five factors associated with PPD were low perceived social support, HIV positive status, rural residence, obstetrical complications and the baby crying excessively.

**Conclusion and recommendations:**

Prevalence of PPD in Mbarara and Rwampara districts is higher than what has previously been reported in Uganda indicating an urgent need to identify pregnant women who are at increased risk of PPD to mitigate their risk or implement therapies to manage the condition. Midwives who attend to these mothers need to be empowered with available methods of mitigating prevalence and consequences of PPD. Women who are HIV positive, residing in rural settings, whose babies cry excessively, having low social support systems and who have birth complications may be a particularly important focus for Ugandan intervention strategies to prevent and reduce the prevalence of PPD.

**Supplementary Information:**

The online version contains supplementary material available at 10.1186/s12884-021-03967-3.

## Background

Postpartum depression (PPD) is a significant cause of maternal morbidity and mortality [1–3]. It also has severe consequences on the physical and social well-being of mothers, new-born infants, families and communities. PPD reduces the mother’s response to the child’s needs and in severe cases, mothers suffering from PPD are prone to postpartum psychosis, suicide and rarely infanticide [4–6]. However, research on PPD has been given minimal attention in low-income countries. Postpartum depression is a non-psychotic disabling mood disorder occurring in the first 6 weeks following childbirth [7, 8]. During the postpartum period, the woman is expected to recover from the stress of pregnancy, birth and physiological adaptation after childbirth. PPD is characterized by severe mood swings, loss of interest, sleep disturbances, feelings of sadness, excessive crying, fatigability, appetite loss, coping stress with daily activities and thoughts of self-harm or harm to the baby [9]. If risks for PPD are detected early, PPD is preventable and treatable using less risky non-pharmacological methods [10]. Globally, it is estimated that 10 to 15% of women suffer from PPD [11, 12]. High-income countries have a lower prevalence of PPD compared to low-income countries.

In low-income countries such as Uganda, determining the actual prevalence of postpartum depression remains problematic due to limited screening, diagnosis and under-reporting by postpartum care providers. Although studies on PPD in Africa are limited, available studies report varying prevalence from 6.9 to 39.6% [12–14]. Most of these studies used screening tools to identify PPD rather than reliable diagnostic criteria [15].

In Uganda, there is paucity of data regarding the prevalence of PPD. A PPD prevalence of 6.1% was reported in a peri-urban setting and in a study that utilized the Self Reporting Questionnaire (SRQ-20) tool [16]. Another study using the Edinburgh Postnatal Depression Scale (EPDS) reported a prevalence of 43% in rural Uganda [17]. There is no documented Ugandan study that screened and clinically confirmed PPD.

PPD is associated with socio-demographic, psychosocial, obstetric factors, and influenced by culture in low income countries [13, 18]. This paper is a step towards understanding factors associated with PPD in the cultural context of Uganda. Determining the prevalence and understanding factors associated with PPD is fundamental to inform policy makers and prepare health care workers (HCW) to implement PPD assessment to conform with Ugandan Clinical Guidelines [19]. Failure to assess and manage PPD has adverse implications towards Uganda’s efforts to meet the Sustainable Development Goals (SDG) and the national development targets. Failure to do so has poor mental health effects on the productivity of affected women and their households [19]. Preventing the disorder and promoting mental health has positive implications as compared to treatment of PPD. This study sought to determine prevalence of PPD and factors associated with PPD in Mbarara and Rwampara districts, southwestern Uganda.

## Methods

### Study design

This paper presents a multi-facility, cross-sectional study that gathered data from mothers during their 6 to 8 weeks’ postpartum periods in Mbarara and Rwampara districts, southwestern Uganda.

### Study setting

This study was conducted in Mbarara Regional Referral Hospital (MRRH), Bwizibwera Health Centre IV (BHC-IV) and Kinoni Health Centre IV (KHC-IV), approximately 270 km from Kampala, the capital city of Uganda. Mbarara Regional Referral Hospital (MRRH) is in the urban setting (Mbarara city) and the two HC-IVs, KHC-IV in Rwampara district and BHC-IV in Mbarara district in the rural setting, both about the same distance from MRRH. These facilities were selected because most births take place there owing to fully functioning theatres that support any mode of birth. Municipal HC-IV was not selected because it had been used for pre-testing the study tools.

MRRH is a referral facility for the southwestern region providing both in-patient and out-patient care. It has eight main departments: mental health, medical, surgical, pediatrics, obstetrics and gynecology, theatre, intensive care unit and outpatient departments, including maternal and child health (MCH). The postnatal clinic has ten health workers and an average daily attendance of 30 mothers. Care includes immunization, family planning, health education, infant growth monitoring and examination of postpartum mothers.

At the time of the study, BHC-IV had eight midwives and an average daily attendance of 16 mothers and KHC-IV had four midwives and an average of 30 mothers who attended the clinic daily, plus approximately 6 births recorded daily.

### Study population and period

Data was collected from 11^th^ November 2019 to 10^th^ June 2020. The study targeted mothers who visited the postnatal clinic for their routine 6-week postpartum visit according to the Ugandan MOH Guidelines [19]. Sample size was determined using a single population formula with correction for finite population [20]. That is:

- *n* = N*X / (X + N – 1), Where,
- X = Zα/22 ¬*p*(1-*p*) / MOE2,
- Zα/2 is the critical value of the normal distribution at α/2 for α of 0.05 = 1.96,
- MOE is the margin of error considered at 5%,
- *p* = expected proportion of mothers with diagnosis of PPD,
- *N* is the population size of mothers who delivered at the selected study sites over the recent 3-month period.

The minimum sample size targeted was 231 mothers attending post-natal clinics in the study sites. With additional 20% to cater for attrition, the final sample size was 292 mothers.

### Sampling procedure

Using proportionate stratified consecutive sampling, the sample size was divided with the health facility as a stratum. This was based on the number of women giving birth in each health facility the previous 3 months. The probability proportionate to size was used to determine the number of mothers that were enrolled at each stratum to meet the calculated sample size of 292. Mothers within each health facility were selected consecutively based on the eligibility criteria until the sample size apportioned for each stratum had been attained.

### Inclusion and exclusion criteria

Women were considered eligible if they were between 18 and 49 years old and attended the clinic during the 6^th^ to 8^th^ week postpartum in the post-natal clinic. Mothers were excluded, if they reported to the clinic for illness with complaints of high-grade fever, high blood pressure, vaginal bleeding or were unable to respond to the questions. In MRRH, mothers were also excluded if they were residing outside the boundaries of Mbarara city.

### Study variables

Main outcome measure was PPD among mothers. Women were classified as PPD if they had positive DSM-V criteria [21]. This was a binary variable coded 0 for PPD absent and 1 for PPD present. Independent variables were socio-demographic characteristics, maternal obstetric and medical factors, baby factors and social support factors.

### Measurements

**Maternal age** was reported by the women at the time of data collection and grouped into four categories: younger than 20 years for adolescents, 20 to 24 years for young mothers, 25 to 34 years for middle reproductive and 35 to 49 years for older reproductive age.

**Parity** is the number of pregnancies from 28 weeks.

**Residence** was classified as either rural or urban. For this study, urban was defined as people who were residents of Mbarara city with good accessibility to health care, road and commercial settings; while rural areas have a low population density and large amounts of undeveloped land [22, 23].

**Housing** was classified as either temporary (grass-thatched houses) or permanent (brick-built houses with iron sheets).

**Obstetric complications** are birth complications the mother had while giving birth, listed as excessive bleeding, obstructed labor, infection or high blood pressure.

**Maternal medical history** is the state of the mother at the time of birth. The factors collected were HIV positive or not and other chronic conditions like diabetes mellitus, hypertension or any others.

**Occupation** was referred to as how people earned a living, ranging from being unemployed to being employed as a professional or laborer according to Uganda Bureau of Statistics [24, 25].

**Social support** is support respondents receive from spouses, family members, and friends. This was measured by the Maternal Social Support Scale (MSSS) [26]. This validated tool measures perceived social support from family, partners, and friends. Participants are asked the extent of support from their significant others’ on a 5-point Likert scale, where 1 is never and 5 always. Scoring for two items is reversed. Scores range from 0 to 30, categorized as low (0–18), medium (19–24) and adequate (> 24) [26]. Cronbach’s alpha value for this tool was documented as between 0.71 and 0.90 when applied to Syrian women in Jordan [27].

**Baby related factors** were the fetal factors in the current delivery that were associated with PPD. Baby related factors includes the sex of the baby, complications at birth, delivered baby at 9 months, baby having health problems them, baby who was hospitalized in the last 6 weeks, baby breast feeding well and problems with baby sleeping. The factors were recorded as a Yes/No response.

**Baby crying extensively** is the baby crying more excessive than normal although this information was subjectively reported by the mother and recorded as a Yes/No response.

### Data collection tools

This study used a Diagnostic and Statistical Manual of Mental Disorders, 5th Edition (DSM-V) to determine the prevalence of PPD. We also used a validated structured interviewer administered questionnaire to gather data about participants’ characteristics to determine possible factors associated with PPD. The questionnaire had four sections with the following content: socio-demographic data, baby-related factors, maternal obstetric and medical factors and the final section contained the social support factors. These questions were adopted from earlier studies [28].

### Data collection and data quality assurance

The English version of the questionnaire (see supplementary file) was translated to the local language (Runyankore-Rukiga) and back to English to check for consistency and accuracy. Four graduate nurses, one diploma midwife, one psychiatric clinical officer and two psychiatrists were trained as research assistants and clinicians for the three study sites. The first author and co-authors closely managed the data collection process. Training of overall content of the tools and data collection processes was conducted over five consecutive days for data collectors. We pre-tested the tools on 5% of the calculated sample size of postpartum mothers in Municipal HC-IV and revisions made as necessary to improve on contextual validity.

After consent and enrollment of eligible postpartum mothers in each site, they were first subjected to the questionnaire by nurses or midwives. All the participants were then moved to another private room where they were clinically examined by a psychiatrist using DSM-V to establish presence or absence of PPD.

### Data analysis

Collected data were checked manually for completeness and consistency to minimize data entry errors. Coded data were then entered into EPI Info software version 7.2. Appropriate data validation and cleaning was done prior to exporting the database for analysis. The dataset was then imported into STATA software version 13∙0 for analysis (Stata Corp, College Station, TX, USA).

Participants’ characteristics were described using proportions for categorical variables. Prevalence of PPD was calculated as a proportion of postpartum mothers diagnosed with PPD using DSM-V criteria out of all mothers enrolled into the study and expressed as a percentage with corresponding 95% confidence interval (CI). Prevalence was also expressed and compared by site, age categories and parity using Chi-square test. Statistical significance was achieved when *p* ≤ 0.05.

The dependent variable was the dichotomous presence or absence of PPD. The bivariate analyses used Chi-square exact test to determine correlations between each independent variable with the dependent variable. Unadjusted odds ratios with their corresponding 95% CI are reported.

The independent variables in bivariate analysis with *p*-values < 0.1 were included in multivariate logistic regression analysis. Absence of multicollinearity among independent variables was confirmed [29]. A manual back-ward stepwise selection method was used in the final multivariate analysis with factors bearing independent significant associations with PPD. The goodness-of-fit test was performed on the final model to assess its quality. Factors in the final multivariate model are reported together with their adjusted odds ratios (aORs) and 95% CI.

## Results

### Participant characteristics

Of the total of 580 women who were approached between November 2019 and June 2020, 275 were ineligible to participate for being less than 6 weeks and more than 8 weeks postpartum. Seventeen potential participants declined participation due to time constraints (11) and having ill children (6). There were 292 postpartum mothers who consented and enrolled into the study, with 152 enrolled from MRRH, 93 from BHC-IV and 47 from KHC-IV.

#### Sociodemographic characteristics

The majority of mothers were between 20 and 34 years (80.1%), were married (95.2%), identified as Banyankore by tribe (76.4%), had attained at least secondary school education (36.3%), were residing in urban areas (66.1%) and were dwelling in permanent houses (79.5%) (Table 1).

**Table 1:** Sociodemographic, maternal obstetric/medical and bivariate analysis of factors associated with postpartum depression

Variable	*n* (%)	Depressed	Not Depressed	Unadjusted OR (95% CI)	*P-value*
		No, *n* (%)	Yes, *n* (%)		
**Social-Demographic Factors**					
Health facility					0.260
Bwizibwera	93 (32.8)	63 (67.7)	30 (32.3)	1.0	
Kinoni	47 (16.1)	33 (70.2)	14 (29.8)	0.9 (0.42 – 1.91)	
MRRH	152 (52.1)	117 (77.0)	35 (23.0)	0.6 (0.35 – 1.12)	
^c^Residence					0.005
Rural	99 (33.9)	62 (62.6)	37 (37.4)	2.1 (1.26 – 3.65)	
Urban	193 (66.1)	151 (78.2)	42 (21.8)	1.0	
Age (years)					0.175
< 20	16 (5.5)	8 (50.0)	8 (50.0)	3.4 (1.16 – 10.16)	
20–24	102 (34.9)	79 (77.5)	23(22.6)	1.0	
25–34	132 (45.2)	95 (72.0)	37 (28.0)	1.3 (0.73 – 2.44)	
35–49	42 (14.4)	31 (73.8)	11 (26.2)	1.2 (0.53 – 2.79)	
Tribe					0.155
Munyankore	223 (76.4)	159 (71.3)	64 (28.7)	1.0	
Muganda	18 (6.2)	11 (61.1)	7 (38.9)	1.6 (0.59 – 4.26)	
Mukiga	31 (10.6)	26 (83.9)	5 (16.1)	0.5 (0.18 – 1.30)	
Others	20 (6.8)	17 (85.0)	3 (15.0)	0.4 (0.12 – 1.55)	
Education					0.281
None	4 (1.4)	2 (50.0)	32 (33.0)	1.0	
Primary	97 (33.2)	65 (67.0)	25 (23.6)	0.5 (0.67 – 3.66)	
Secondary	106 (36.3)	81 (76.4)	20 (23.5)	0.3 (0.41 – 2.31)	
Tertiary	85 (29.1)	65 (76.5)	79 (27.1)	0.3 (0.41 – 2.33)	
Marital status					0.467
Unmarried	14 (4.8)	9 (64.3)	5 (35.7)	1.0	
Married	278 (95.2)	204 (73.4)	74 (26.6)	0.7 (0.21 – 2.01)	
Religion					0.415
Catholic	93 (31.8)	2 (50.0)	2 (50.0)	1.0	
Protestant	145 (49.7)	65 (67.0)	65 (67.0)	0.9 (0.52 – 1.73)	
Moslem	24 (8.2)	81 (76.4)	81 (76.4)	1.2 (0.44 – 3.20)	
Others	30 (10.3)	65 (76.5)	65 (76.5)	1.9 (0.81 – 4.55)	
Occupation					0.096
Unemployed	60 (20.5)	46 (76.7)	14 (23.3)	1.0	
Business	114 (39.0)	87 (76.3)	27 (23.7)	1.0 (0.49 – 2.13)	
Subsistence farmer	57 (19.5)	35 (61.4)	22 (38.6)	2.1 (0.93 – 4.60)	
Professional	51 (17.5)	40 (78.4)	11 (21.6)	0.9 (0.37 – 2.21)	
Manual labourer	10 (3.4)	5 (50.0)	5 (50.0)	3.3 (0.83 – 13.01)	
Housing					0.127
Temporary	232 (79.5)	174 (75.0)	58 (25.0)	1.0	
Permanent	60 (20.5)	39 (65.0)	21 (35.0)	1.6 (0.88 – 2.97)	
**Maternal Medical Factors**					
^c^HIV status					0.002
Positive	51 (17.5)	28 (54.9)	23 (45.1)	2.7 (1.45 – 5.08)	
Negative	241 (82.5)	185 (76.8)	56 (23.2)	1.0	
^c^Known HIV status of husband^b^					0.042
No	33 (11.3)	19 (57.6)	14 (42.4)	2.2 (1.04 – 4.63)	
Yes	259 (88.7)	194 (74.9)	65 (25.1)	1.0	
Chronic medical conditions					0.193
No	272 (93.2)	201 (73.9)	71 (26.1)	1.0	
Yes	20 (6.8)	12 (60.0)	8 (40.0)	1.9 (0.74 – 4.81)	
Number of children					0.380
1	108 (37.0)	81 (75.0)	27 (25.0)	1.0	
2–4	152 (52.1)	112 (73.7)	40 (26.3)	1.1 (0.61 – 1.89)	
5 or more	32 (11.0)	20 (62.5)	12 (37.5)	1.8 (0.77 – 4.16)	
Deceased children					0.407
None	261 (89.4)	188 (72.0)	73 (28.0)	1.0	
Less than half	16 (5.5)	12 (75.0)	4 (25.0)	0.9 (0.27 – 2.75)	
More than half	15 (5.1)	13 (86.7)	2 (13.3)	0.4 (0.09 – 1.80)	
Birth place recent pregnancy^a^					0.255
Health facility	283 (96.9)	208 (73.5)	75 (26.5)	1.0	
Not health facility	9 (3.1)	5 (55.6)	4 (44.4)	2.2 (0.58 – 8.48)	
^c^Complication in recent pregnancy					0.015
No	238 (81.5)	181 (76.1)	57 (24.0)	1.0	
Yes	54 (18.5)	32 (59.3)	22 (40.7)	2.2 (1.18 – 4.05)	
Mode of birth in recent pregnancy					0.959
Vaginal birth	236 (80.8)	172 (72.9)	64 (27.1)	1.0	
Caesarian section	56 (19.2)	41 (73.2)	15 (26.8)	1.0 (0.51 – 1.90)	

^a^Birth with a skilled health worker is not expected in home birth

^b^Father of the baby

^c﻿^Factors significant at bivariate analysis

#### Obstetric and medical characteristics

The majority of participants had given birth to at least two children prior to the current pregnancy (63.1%) and had not experienced previous child deaths (89.4%). In the current pregnancy, most mothers (96.9%) gave birth in health facilities with skilled health workers and by vaginal route (80.2%). Overall, 17.5% were HIV positive and 88.7% were aware of the HIV status of their spouses (Table 1).

#### Baby-related characteristics

Most babies were male (51.4%) and 10.3% had complications or abnormalities at birth. Key complications included infections (6.7%), jaundice (30%), birth asphyxia (23.3%), congenital anomalies (16.7%), and (10.3%) preterm birth. Health problems after birth included 9.3% of babies who had been hospitalized in the prior 6 weeks, 3.8% of babies were not breastfeeding well, 33.9% had sleeping problems, 22.6% cried excessively, and 20.9% were difficult to console (Table 2).

**Table 2:** Baby factors, social support characteristics and bivariate analysis of factors associated with postpartum depression

Variable	*n* (%)	Depressed	Not Depressed	Unadjusted OR (95% CI)	*P-value*
		No, *n* (%)	Yes, *n* (%)		
**Baby- Related Factors**					
Sex of the baby					0.523
Male	150 (51.4)	107 (71.3)	43 (28.7)	1.0	
Female	142 (48.6)	106 (74.7)	36 (25.4)	0.8 (0.50 – 1.42)	
Abnormality/complications at birth					0.103
No	262 (89.7)	195 (74.4)	67 (25.6)	1.0	
Yes	30 (10.3)	18 (60.0)	12 (40.0)	1.9 (0.89 – 4.24)	
Experiencing health problem now					0.351
No	253 (86.6)	187 (73.9)	66 (26.1)	1.0	
Yes	39 (13.4)	26 (66.7)	13 (33.3)	1.4 (0.69 – 2.92)	
Birth at term (9 months)					0.959
No	30 (10.3)	22 (73.3)	8 (26.7)	1.0	
Yes	262 (89.7)	191 (73.0)	71 (27.1)	1.0 (0.44 – 2.40)	
Baby hospitalized last 6 weeks					0.889
No	265 (90.8)	193 (72.8)	72 (27.2)	1.0	
Yes	27 (9.2)	20 (74.1)	7 (25.9)	0.9 (0.38 – 2.31)	
^b^Baby breastfed well					0.049
No	11 (3.8)	5 (45.5)	6 (54.6)	3.4 (1.01 – 11.54)	
Yes	281 (96.2)	208 (74.0)	73 (26.0)	1.0	
Problems with baby sleeping					0.150
No	193 (66.1)	146 (75.7)	47 (24.4)	1.0	
Yes	99 (33.9)	67 (67.7)	32 (32.3)	1.5 (0.87 – 2.53)	
^b^Baby crying excessively					0.012
No	226 (77.4)	173 (76.6)	53 (23.5)	1.0	
Yes	66 (22.6)	40 (60.6)	26 (39.4)	2.1 (1.19 – 3.80)	
Baby difficult to console					0.423
No	231 (79.1)	171 (74.0)	60 (26.0)	1.0	
Yes	61 (20.9)	42 (68.9)	19 (31.2)	1.3 (0.70 – 2.39)	
**Social Support Factors**					
^b^Perceived social support		^a^			0.016
Low	44 (15.1)	25 (56.8)	19 (43.2)	3.4 (1.44 – 8.12)	
Medium	182 (62.3)	134 (73.6)	48 (26.4)	1.6 (0.79 – 3.27)	
Adequate	66 (22.6)	54 (81.8)	12 (18.2)	1.0	

^a^Includes support from friends, family and husband and scored as Low (0–18), Medium (19–24), Adequate (> 24)

^b^Factors significant at multivariate analysis

#### Social support among participants

Most mothers experienced medium social support (62.3%), 15.1% perceived low social support from husband/partners, family, and friends and 22.6% had adequate social support. Types of support provided by spouses included helping with household chores (72.3%), babysitting (78.1%), financial support that paid for clinic visits (91.4%), nutritional support (90.8%) and counseling support (83.2%). Most mothers (71.3%) were aware of the condition of PPD (Table 2).

### Prevalence of PPD

The overall prevalence of PPD was 79/292 (27.1%; 95% CI 22.2–32.5). On stratification by health facility, BHC-IV had the highest prevalence of 30/93 (32.3%; 95% CI 23.4–42.6) as compared to 14/47 (29.8%; 95% CI 18.1–44.8) in KHC-IV and 35/152 (23%; 95% CI 17.0–30.5) in MRRH, however this difference in prevalence between the study sites was not statistically significant. The prevalence of PPD did not vary in a statistically significant way by maternal age, the number of previous births or mode of delivery.

### Factors associated with PPD

The only sociodemographic factor with an association to PPD was place of residence. Associated maternal medical factors were HIV positive serology status of the mother, unknown HIV serology status of the husband and birth complications in recent pregnancy (Table 1). Factors related to the baby were not breast feeding well, a baby who cried excessively and perceived social factors. In total, the chi-square bivariate analysis revealed seven factors with statistically significant associations to PPD. Occupation was included in the logistic regression model because the *p*-value was less than 0.1 making a total of eight factors (Table 1).

### Multivariate regression analysis

The overall mean variance inflation factor (VIF) for all independent variables together was 1.21, meeting assumptions for logistic regression modeling [29]. The non-significant goodness – of – fit test based on the Hosmer and Lemeshow chi-square indicated a good fit of the model (*p* = 0.28) [30]. The classification table indicated the model improved the ability to accurately predict PPD outcome from 27% (null model without predictors) to 74% with the inclusion of the predictor variables.

In the final model, five variables demonstrated statistically significant predictive values for PPD. These were perceived social support, HIV positive serology status of the mothers, rural residence, any complication in recent pregnancy and a baby who cried excessively (Table 3).

**Table 3:** Results of multivariate analysis for factors independently associated with PPD

Variable	Adjusted OR (95% CI)
^a^Residence	
Rural	1.9 (1.03 – 3.64)
Urban	1.0
Occupation	
Unemployed	1.0
Business	1.1 (0.89 – 2.36)
Peasant farmer	1.4 (0.57 – 3.48)
Professional	1.3 (0.49 – 3.42)
Manual labourer	2.3 (0.51 – 10.62)
Baby breastfeeding well	
No	3.4 (0.94 – 12.15)
Yes	1.0
^a^Baby cries excessively	
No	1.0
Yes	2.2 (1.15 – 4.08)
^a^HIV status	
Positive	2.1 (1.04 – 4.17)
Negative	1.0
Known HIV status of husband	
No	1.0
Yes	1.7 (0.71 – 3.85)
^a^Any complication in recent pregnancy	
No	1.0
Yes	2.0 (1.00 – 3.85)
^a^Perceived social support	
Low	2.3 (0.87 – 6.17)
Medium	1.3 (0.62 – 2.85)
Adequate	1.0

^a^Model characteristics: *N* = 292; χ^2^ (11) = 38.54, *p* < .001, Pseudo-*R*^2^: .1130

## Discussion

### Prevalence of PPD

The prevalence of postpartum depression among the 292 mothers was 27.1%, much higher than 6.1% and lower than 43%, reported by previous Ugandan studies [16, 17]. It was also higher than the prevalence reported in other African countries such as Ethiopia (20.9–22.9%), Sudan (9.2%), Kenya (13–18.7%), Tanzania (12%) and Ghana (7%) [15].

The prevalence is, however, lower than the range of 31.7 to 50.3% reported from South Africa and 33% in Zimbabwe [31]. The variance between our study and the other studies can be attributed to the variations in sample sizes, tools used to measure PPD, using a screening tool as a diagnostic tool without validating the tool, timing of postpartum period and socio-cultural differences. For instance, in a peri-urban health centre in Kampala women were assessed at 6 weeks postpartum and prevalence of PPD was determined using a 25-Item Self-Reporting Questionnaire (SRQ-25) and MINI to confirm major depression [16]. MINI, however, was more inferior than DSM-Vin diagnosing for PPD since it broadly assessed for major depression and this study was implemented 15 years ago. Other reports of much higher prevalence in southwestern Uganda may be attributed to use of the untranslated EPDS screening tool used with mothers who may have limited understanding of English [17]. Further, the EPDS was administered as a diagnostic tool instead of screening, for which it was originally designed. Use as a diagnostic tool without adaption to the local context, is likely to over-estimate or under-estimate the burden and prevalence of PPD. In our study, the prevalence was determined by clinically diagnosing mothers with DSM-V, which is the “gold standard” diagnostic criterion. These diagnostic tools were translated to the local language, Runyankore, for participants with limited English proficiency.

### Factors associated with PPD

Mothers’ perceived social support by husbands/partners, family and friends; HIV positive status of the women; babies who cried excessively; women with complications in the current pregnancy; and rural residence were significantly associated with a clinical diagnosis of PPD. Social support is important during and after childbirth which is in agreement with studies indicating social support as a protective factor among women with PPD [32]. In rural South Africa, a rate of 60.9% of mothers without family or social support suffered from severe depression as compared to 28.2% who had moderate depression [32]. Several studies from Cameroon, Ethiopia and Pakistan identified lack of social support as a risk factor for PPD [33–36].

During the post-partum period women should be recovering from the stress of pregnancy, birth and physiological adaptation after childbirth. To reduce this postpartum related stress, mothers are advised to receive social support from their husbands/partners, family relations and friends. Social support can prevent PPD and its after effects, optimizing mothers’ positive self-images and enhancing quality of life [37].

There is scanty literature reporting the association between new-borns who cry excessively and PPD. Participants who reported their babies cried excessively in present study were 2.2 times more likely to experience PPD. If babies cry much and women receive no or low social support from husbands/partners, they may be deprived of sleep leading to PPD [38]. Babies who cry excessively may not be breastfed well and this causes anxiety in the mothers, hence potentially contributing to PPD development [39, 40]. PPD adversely influences infant development and behaviour such as crying and breast feeding, but it remains unclear if this correlation is causal and if so, in what direction [41]. Other behaviour influenced by PPD includes irritability and difficulty in soothing and consoling the baby.

HIV-positive participants in the present study were 2.1 times more likely to experience PPD than HIV-negative ones. Other studies in similar settings have reported an increased risk of PPD among HIV-infected mothers, consistent with our study [42–44]. Women living with HIV/AIDS are at high risk of PPD, because they face several risk factors like lack of social support, stigma and discrimination [45].

The study setting may have an effect on the correlation between HIV status and PPD. Research from high income countries predominantly report no association between HIV and PPD prevalence [46–48]. This lack of association could be explained by the type of care provided to postpartum mothers following childbirth. Mothers in high income countries may be more likely to receive systematic follow up and counseling from nurses and social workers in accordance with local government health systems than mothers in low-resourced settings. The children born to mothers in high income countries are less likely to be HIV positive because of the structured Prevention of Mother To Child Transmission (PMTCT) programs which reduce transmission and incidence of newborn babies with HIV [49]. A significant risk for PPD was, however, reported in HIV-positive mothers whose infants were also diagnosed with HIV [50]. This calls for integration of psychosocial support in the postpartum care given to HIV positive women.

Mothers with a history of any birth complications in the most recent pregnancy were twice more likely to suffer from PPD as compared to those without complications. Each year in Africa, 30 million women become pregnant and about 250,000 of them die from pregnancy-related causes. Several studies identified a strong association between obstetric complications and PPD [51–54]. A study delineating the association between heavy postpartum haemorrhage and PPD in Uppsala, Sweden observed direct and positive influences between anaemia at discharge from hospital and self-reported bad birth experiences with PPD [52]. Anaemia is a consequence of excessive vaginal bleeding or postpartum haemorrhage and mothers are likely to present with fatigue, reduced cognitive abilities, emotional disturbances and consequently PPD [55]. When these mothers are distressed, they are likely not breastfeeding well, hence their babies may be more apt to cry excessively.

Pre-eclampsia has also been shown as a risk factor to PPD [56]. Serotonin levels in blood are known to be high in women suffering from hypertension [57]. This may lead to decreased serotonin levels in the mothers’ brain, thereby causing PPD. Women who experience birth complications are likely to experience physical and psychological morbidity after birth [58].

Participants in the present study who live in rural areas, were twice more likely to experience PPD compared to those living in urban areas. The impact of place of residence has not been adequately studied on risk of suffering from PPD in low-resourced settings. However, studies from middle income countries, such as Bangladesh, India, Iran and Pakistan concur with our findings that PPD is more prevalent in mothers residing in rural settings [59–63]. Rural Ugandan women may be more at risk of suffering from PPD because they mostly depend on subsistence agriculture with low standards of living and are commonly poorer than women in urban settings [22, 25].

One study reported no association between residing in urban or rural areas and PPD [64]. Conversely in high-income countries like Canada and Australia higher rates of PPD are reported among mothers in urban settings than those in rural areas [65–67]. This is possibly due to geographical differences between urban settings in high-income and low-income countries. More frequently in high-income countries, urban settings may be associated with dense populations, higher rates of interpersonal violence, poorer health and lower social support as compared to rural settings [65].

### Strengths and limitations

A key strength was the use of a “gold standard” DSM-V to clinically diagnose and confirm mothers suffering from PPD.

This study was limited to women having given birth in public health facilities, hence results should cautiously be generalized to private health facilities, given the expected differences in socio-demographic and economic characteristics of patients that visit these two settings. Previous pregnancy experience was not the focus of this study whereas some literature highlights a strong association between previous birth experience and pregnancy related variables. In addition, the cross-sectional design limits the capacity to draw any causal implications especially between some variables and PPD, given its limitation in establishing temporal relationships between independent variables and outcome [68]. The fact that two-thirds of the participating mothers were from urban settings with good access to health facilities could have resulted in an underestimate of prevalence of PPD. This study was carried out during the global pandemic of COVID-19 that may have increased PPD prevalence in Uganda. Despite these limitations, this is the first study in the Uganda setting that clinically diagnosed mothers with PPD in rural and urban public health facilities. Our study findings are useful to inform the magnitude and risk factors associated with PPD in Uganda for planning and integrating PPD prevention and care to women at risk.

### Implications and recommendations

These findings can be useful for practice and policy in Uganda and other low-resource settings. They point out the need to increase public awareness about PPD and its associated factors. The high prevalence of PPD among Ugandan women indicates an urgent need to identify pregnant women at increased threat of PPD to mitigate the risk or implement therapies to manage the condition. Because of the high prevalence, PPD screening and intervention need to be integrated with maternal health care in public health facilities, especially for mothers at risk of PPD. In addition, PPD burden needs to be evaluated in mothers attending private health facilities so as to guide the scaling of appropriate interventions to these facilities. Midwives who attend to these mothers should be trained to screen for PPD.

Such training would require development of educational initiatives to disseminate the risks for PPD and adverse consequences of unidentified and untreated PPD. There is a need to validate a culturally and linguistically relevant screening tool that can be applied by all nurses and midwives for PPD. Validation of the EPDS screening tool through translation and establishment of culturally appropriate cut-off point for optimal sensitivity and specificity has been implemented in another arm of this current study. Results of this validation will be reported elsewhere. Inclusion of such a validated screening tool within the Uganda Clinical Guidelines will empower Ugandan midwives to practice at their highest potential to mitigate the prevalence and consequences of PPD.

Women who are HIV infected, reside in rural settings, whose babies cry excessively, have low social support and who have birth complications may be a particularly important focus for Ugandan intervention strategies to prevent and reduce PPD. Mothers’ perceptions of adequate social support and their relationships with their relatives and friends are potential facilitators to reduce burden of PPD. This strength should be considered in development of community interventions to benefit pregnant women, focusing on improving family and community relationships.

These findings are indicative of the need for midwives and nurses working in rural areas to ensure comprehensive assessment of postpartum mothers, most especially those living with HIV to offer the best chance of implementing holistic care for postpartum women suffering from PPD.

Future studies may utilize longitudinal methods to track a single group of mothers to examine causal relationships between depression and risk factors identified in this study. Although various socio-demographic, maternal obstetric medical, baby related and social support correlates were examined in this study, prior research has shown that biological factors such as oxytocin levels and personality traits are also significantly correlated with PPD. More personal and biological predictors could be examined in future research.

## Conclusion

More than one-quarter of the women in this study demonstrated clinical diagnosis of PPD, indicating a high prevalence in south-western Uganda. Whereas the most recent HIV-prevalence in south-western Uganda stands at 7.9%, comparable to 8% in the whole of Uganda, PPD is more prevalent among HIV-positive mothers and residing in rural settings [25]. Addressing PPD in this context, nurses in postnatal clinics should tailor interventions and target HIV-positive mothers, those with low social support or a history of birth complications, having babies that cry excessively, or reside in rural settings. Nurse-midwives in maternal health should be aware of PPD and be able to provide immediate holistic care.

This study provides an important contribution to the fight against maternal mental illness and its associated factors. A qualitative study to understand the lived experiences, challenges and coping mechanisms can be done in this health district.

## Supplementary Information

**Additional file 1.**

## Data Availability

The data that support the findings of this study are available from the first and corresponding author, Catherine Atuhaire but restrictions apply under license for the current study.

## Abbreviations

BHC-IV: Bwizibwera Health Centre IV
CI: Confidence interval
DSM-V: Diagnostic and Statistical Manual of Mental Disorders, 5th Edition
EPDS: Edinburgh Postnatal Depression Scale
HCW: Health care workers
KHC-IV: Kinoni Health Centre IV
PPD: Postpartum depression
SRQ: Self-Reporting Questionnaire (SRQ-20)
SDG: Sustainable Development Goals
HIV: Human Immunodeficiency Virus
MCH: Maternal and child health
MRRH: Mbarara Regional Referral Hospital
MSSS: Maternal Social Support Scale
