# Supplementary material for: Prevalence of postpartum depression and associated factors among women in Mbarara and Rwampara districts of south-western Uganda
Source: BMC Pregnancy Childbirth. 2021 Jul 12;21:503. doi: 10.1186/s12884-021-03967-3 (PMC8272966; doi:10.1186/s12884-021-03967-3)
Supplement: Supplementary file 1 — Additional file 1. [file 12884_2021_3967_MOESM1_ESM.docx]

March 16, 2021

**BMC Pregnancy and Childbirth**

Dear Maggie,

**Re: Resubmission of manuscript ‘PRCH-D-20-02430-Prevalence of Postpartum Depression and Associated Factors among women in Mbarara and Rwampara Districts of South Western Uganda’**

Thank you for giving us an opportunity to resubmit our paper to BMC Pregnancy and Childbirth after revising it following the review comments. The comments are greatly appreciated and have been utilized to improve the manuscript as indicated in the revised version. Here below are the point by point responses to the review comments:

| **Section** | **Comments** | **Authors’ response** |
| --- | --- | --- |
| **Reviewer 1** | | |
| **Introduction** | 1.      The statement (lines 8/9) that postnatal depression has been neglected in the literature is probably not the case, in fact you cite many studies in the discussion; 2.      The use of terms like developed/ developing is somewhat dated; I suggest using high income/ low income; | 1. Thank you for the observation. The statement has been revised to remove the word ‘neglected’. (See line 8-9).  2. The terms like developed / developing have been replaced with high / low-income countries as suggested by the reviewer. |
| **Methods** | 3. The presentation of the sample size calculations is very technical and could be rewritten using a more standard approach such as specifying the hypothesised prevalence etc;  4. The explanation about the sampling frame could be more streamlined and important information is not evident, namely the stratification variable(s);  5. The section about postpartum depression classification is underdeveloped - more information is required about who and how the assessment was made as well as the cut-points that were used for classification;  6. Who are the language experts? Why do you refer to the Edinburgh scale if you did not use it?  7. I don't understand why you included only statistically significant variables at the bivariate level in the multivariate analysis; it may have been better to use a theoretical approach where you include variables that previous research/ your own observations say should be included; | 3. Thank you for the comment. We have revised the presentation of the sample size calculations as advised. Kindly check Page 5, line 68-69.  4. The explanation about the sampling frame has been streamlined for more clarity and site best explained by health facility. See line 72-76.  5. Thank you for this observation. To answer the objective of this analysis, we depended on the results of the structured clinical interview using the DSM-V only. So we have eliminated any mention of the EPDS. Please check page 7, line 121-127.  6. The language experts are the individuals with experience of translation of a local language with in the local context. However, this sentence has been revised. The EPDS has been removed from this section.  7. We noted all the variables that were statistically significant (p<0.05) but also variables with p< 0.1 and occupation that showed a strong association although it didn’t attain statistical significance in the bivariate were included in multivariate analysis. (Kindly check page 9, lines 153-156). |
| **Results** | 8. I think you need to present a more complete enumeration of the sample given that the aim is to ascertain prevalence; how many women were approached, consented etc. Do you know how many women were approached? This is important as the sampling frame needs to be very well defined for this research question; if it is not known, it needs some statement in the discussion; the limitations of the cross sectional sample also needs some reference vis a vi the consideration of factors associated with depression;  9. A lot of the details in Table 1 do not need to be repeated in such detail in the text;  10. The use of the term confounding is probably not appropriate in this context; the use of the term predictor is also not appropriate given this is a cross-sectional study; | 8. A more complete enumeration of the sample given has been presented that of the total of 580 women who were approached, 275 were ineligible and seventeen potential participants declined participation. Kindly check line 167-170  9. Thanks for this observation. The information in text has been reduced to minimize this repetition.  10. Thank you for the comment. In the revised manuscript, the terms confounding and predictor have been replaced with ‘associated factors’. |
| **Discussion** | 11. I thought the discussion could be made more interesting in considering the implications of the findings. | The discussion has been improved by adding that the there is need to increase public awareness about PPD and its associated factors and how PPD burden needs to be evaluated in mothers attending private health facilities so as to guide the scaling of appropriate interventions. Kindly check page 12-17. |
| **Reviewer 2** | | |
| **General** | 1. The first is the extreme length of the paper with much repetition. I did extensive editing of the paper, which you can find in the attached file. I managed to reduce the numbers of words from 10431 to 8774 (reduction of almost 16%) and don't think your paper has been amputated beyond its real meaning! | 1. Thank you for editing this manuscript extensively. We have reviewed all the corrections accordingly and the manuscript reads much better. |
| **Methods** | 2. There is a problem with statistics, because in doing bi- or multivariate analysis you have to choose the biggest subgroup as reference, and that has not always been done (see also the attached file)  3. In table one it is correct to report p-values because you use these in order to choose your variables for the multivariate, in table 2, however, it is not needed because 95%CI already indicate whether the findings are statistically significant or not. And don't think that 4 digits after the dot in table 1 makes the data more reliable. | 2. The choice of the reference categories in bivariate and multivariate analysis mainly depended on the literature review and these were categories based on the PPD risk assessment. This was to allow consistency in the interpretation of the measures of association as towards increased risk rather than alternating preventive and risk  3. In table 2, P-values have been deleted as advised and the 4^th^ decimal point has been removed from p-values in table 1. |
| **References** | 4. The references are not according to BMC rules (see attached file) | 4. We have revised the references to suit BMC rules of reducing the author number to the first three if they are more than six and using abbreviations of the journals as we found them in pubmed. Kindly check page 21-24. |
| **Reviewer 3** | | |
| **Methods** | * Page 5 line 79: Information on the power calculation was extensive, however I was unclear as to why an expected prevalence for PPD was set at 43% when the rates previously described in the introduction were often much lower than this.  * Page 7 onwards (study variables). The level of detail for a number of variables described here was very good, however there were a few instances where the description could have been expanded upon to improve clarity.  o Was this the exhaustive list of obstetric complications recorded?  o Did the authors collect any information on prior birth experiences? Parity was recorded, but was there any attempt to record mothers' previous birth mode, presence of any prior complications? If so these need detailing here.  o I believe that the MSSS is referred to elsewhere as the maternity social support scale (please reference upon first use). Could the authors provide further information on this scale; e.g., is this measuring perceived social support, receipt of social support, and on what timeframe?  o I'm unsure why the EPDS was not described as with other standardised measurement tools - and I think this is needed. Similarly, much more information is required about how the authors measured prevalence of PPD using the DSM-V. There are some details presented in the procedure section, but ideally information on all measures used is required in the study variables/materials/measurements section. There is a subheading for 'data collection tools' that appears to fall halfway through the measurements section, and could perhaps be re-positioned. The DSM-V is also missing a citation throughout the manuscript.  o Some more information on how the baby-related factors were recorded would be beneficial too - particularly as this features quite prominently in the results and discussion sections  * Page 9 line 158 onwards: When describing the procedure undertaken, could the authors confirm whether all women who consented to participate also took part in the structured clinical interview with the psychiatrist  o Can the authors provide information on the N/% of women approached but did not consent to participate?  * Page 10 lines 174-175: Please could the authors cite the location of information published elsewhere on the concordance between EPDS and DSM-V criteria. | * This information has been revised. PPD prevalence was set at 43% because only two papers determined the PPD prevalence in Uganda: First was determined fifteen years ago and the other reported it at 43% hence the reason we set it at that. Kindly check page 5   - Yes; this was the list of obstetric complications recorded as guided by the literature review - No; we did not explore previous pregnancy experiences like birth mode as the focus of this study was only on the current delivery/birth. - Maternal Social Support Scale (MSSS) has been cited and referenced and more information has been provided about this scale. Kindly check line 110-111 - Including the EPDS is regretted but as explained in the study variables, PPD was diagnosed using the DSM-V criteria. The EPDS has been eliminated as these results will be presented in another paper. The data collection tools section has been revised as advised. DSM-V has been cited and referenced. Kindly check line 86. - More information has been added on baby related factors. Check line 116-119   *All women who consented to participate also took part in the structured clinical interview and this has been rectified as advised   - N% information has been provided in line 198-201.   *The EPDS was deleted as explained earlier as this information will be published in another paper |
| **Results** | * Page 13 line 249 onwards: Could the authors expand in their description of the factors associated with postpartum depression (page 13, lines 250-261) to refer to the direction/detail of these associations in addition to significance in the analysis. I believe 3 decimal places is standard for p-values.  * Page 13 line 266 onwards: Perhaps information about the assumptions testing for the regression would be better placed in the methods section (under data analysis)  * Clarity on how 'the baby was crying a lot' was measured is needed as it was not clear in the materials section.  * Although the findings re. social support and perceptions of the baby are relevant in their association with PPD, the difficulty is inferring a direction of effect with a cross-sectional design. This needs referring to in the limitations section, and some careful consideration (in terms of what was measured, and when) when reporting the results. | *The description of the factors associated with PPD has been amended. P-value has been modified to 3 decimal places in table 1 as advised  * The information about assumptions have been revised as advised  *Baby crying excessively has been clarified in line 118-119  *The limitations of the cross sectional study that it does not establish a temporal relationship between the exposure and outcome. Therefore, we could not rule out that some of the factors like baby crying excessively, perceived social support maybe actually influenced by PPD and this information regarding this type of design has been included in the limitations section. Kindly check line 327-329. |
| **Discussion** | Page 19 line 417: perhaps refer to more than one quarter (rather than one fourth) | We have revised accordingly, thank you for this correction. |
| **Conclusion** | I think some further consideration is required in line with the conclusions. More information is needed about the sampling strategy and recruitment for the study, as without an indication of uptake it is difficult for a reader to interpret whether this sample of women is representative (or whether there may have been some bias in uptake for participation) which holds further implications for reporting prevalence. It would perhaps be preferable for the authors to conclude the % of women reporting PPD 'in the current sample', without extrapolating this to indicate wider prevalence. | More information about the sampling strategy and recruitment has been added as advised. Please check line 167-170. |
|  | As mentioned previously, a number of the variables identified as associated with PPD could be related in a bi-directional manner particularly given the subjective nature of some variables and the use of self-report to record their presence.  Table 1: the column headings for PPD could be improved for clarity - in relation to depression No/Yes columns. | Thank you for this observation. It has been noted in the limitations section.  Column heading for PPD has been improved. |
